# Oocytes could rearrange immunoglobulin production to survive over adverse environmental stimuli

**DOI:** 10.3389/fimmu.2022.990077

**Published:** 2022-11-02

**Authors:** Yang Wang, Fu-Qiang Luo, Yu-Hao He, Zhi-Xia Yang, Xin Wang, Cong-Rong Li, Bei-Qi Cai, Liang-Jian Chen, Zi-Bin Wang, Cui-Lian Zhang, Yi-Chun Guan, Dong Zhang

**Affiliations:** ^1^ State Key Lab of Reproductive Medicine, Nanjing Medical University, Nanjing, Jiangsu, China; ^2^ Department of Ultrasonography, SIR RUN RUN Hospital Affiliated to Nanjing Medical University, Nanjing, Jiangsu, China; ^3^ Analysis and Test Center, Nanjing Medical University, Nanjing, Jiangsu, China; ^4^ Reproductive Medical Center, Henan Provincial People’s Hospital and Reproductive Medical Center, People’s Hospital of Zhengzhou University, Zhengzhou, Henan, China; ^5^ Center for Reproductive Medicine, The Third Affiliated Hospital of Zhengzhou University, Zhengzhou, Henan, China

**Keywords:** oocytes, immunoglobulin, environmental stimuli, LPS, Csf1, *E. coli*, ssRNA

## Abstract

Immunoglobulins are key humoral immune molecules produced and secreted by B lymphocytes at various stages of differentiation. No research has reported whether immunoglobulins are present in the non-proliferative female germ cells—oocytes—and whether they are functionally important for oocyte quality, self-protection, and survival. Herein, we found that IgG was present in the oocytes of immunodeficient mice; the *IgG-VDJ* regions were highly variable between different oocytes, and H3K27Ac bound and regulated the *IgG* promoter region. Next, *IgG* mRNA and protein levels increased in response to LPS, and this increment was mediated by CR2 on the oocyte membrane. Finally, we revealed three aspects of the functional relevance of oocyte IgG: first, oocytes could upregulate IgG to counteract the increased ROS level induced by CSF1; second, oocytes could upregulate IgG in response to injected virus ssRNA to maintain mitochondrial integrity; third, upon bacterial infection, oocytes could secrete IgG, subsequently encompassing the bacteria, thus increasing survival compared to somatic cells. This study reveals for the first time that the female germ cells, oocytes, can independently adjust intrinsic IgG production to survive in adverse environments.

## Introduction

Immunoglobulins are key humoral immune molecules produced and secreted by B lymphocytes at various stages of differentiation. Another unique feature of immunoglobulins is that, in reaction to immunogens, immunoglobulin genes always rearrange before transcription, leading to their extreme polymorphism (1, 2). Several proteins, such as CTCF (3), RAG1/2 (4, 5), VprBP (6), and NF-kappa B (7), are all essential to this rearrangement. Although immunoglobulins have recently been found to exist in several other types of normal cells, including sperm (8), islet cells (9), hepatocytes (10), umbilical cord endothelial cells (11), and eye cells (12), the functional relevance of IgG is far from clearly understood.

Mammalian oocytes are quite distinct from B lymphocytes in several respects. Each diploid oocyte goes through meiosis to produce three degenerating haploid polar bodies and one haploid mature oocyte, which does not continue to go through mitosis or meiosis; mature oocytes, although not proliferative, can mate with a sperm to resume their capacity for division and regenerate stem cells that can differentiate into any tissue cell. Oocytes store plenty of maternal RNAs and proteins and, before resuming meiosis, are transcriptionally quiescent. The oocyte is thought to be a non-professional phagocyte because the fertilization process is somewhat similar to phagocytosis and there are phagocytosis-promoting receptors on the oocyte membrane, including FcγR, CR-1, CR-3, and C1q-R (13). However, no study has shown that oocytes can perform like B lymphocytes to rearrange and produce immunoglobulin.

However, our previous transcriptome and proteome data on mouse oocytes frequently detected immunoglobulin mRNA or protein fragments, prompting us to speculate that oocytes could transcribe and translate immunoglobulin genes. In the present study, we not only prove that immunoglobulin mRNA and protein exist in the oocytes—we also find that oocytes can rearrange immunoglobulin production to survive adverse environmental stimuli.

## Results

### Immunoglobulins are present in oocytes and increase in response to environmental immunogens

Previous studies have found that immunoglobulins also exist within sperm (8), islet cells (9), hepatocytes (10), umbilical cord endothelial cells (11) and eye cells (12) except within B cells. However, the biological relevance of the immunoglobulin within these non-B cells is not known. Oocytes, a special type of terminally differentiated cells, are physically well-protected from viruses or bacteria because they are surrounded by granular cells and embedded inside ovaries. However, oocytes can be damaged by some extremely adverse conditions, such as severe sepsis (14), severe salpingitis (15), and a cytokine storm during a virus infection (15). Furthermore, *in-vitro* maturation or fertilization greatly increase the risk of oocytes being harmed by an adverse cultural environment, such as a contaminated mineral oil or medium. Thus, if oocytes had the capacity to produce immunoglobulin and increase its expression in response to environmental immunogens, they could have a greater chance of survival than somatic cells. Moreover, we frequently saw the presence of immunoglobulin mRNA or protein fragments in ovary transcriptomic or proteomic sequencing databases.

**Figure 1:**
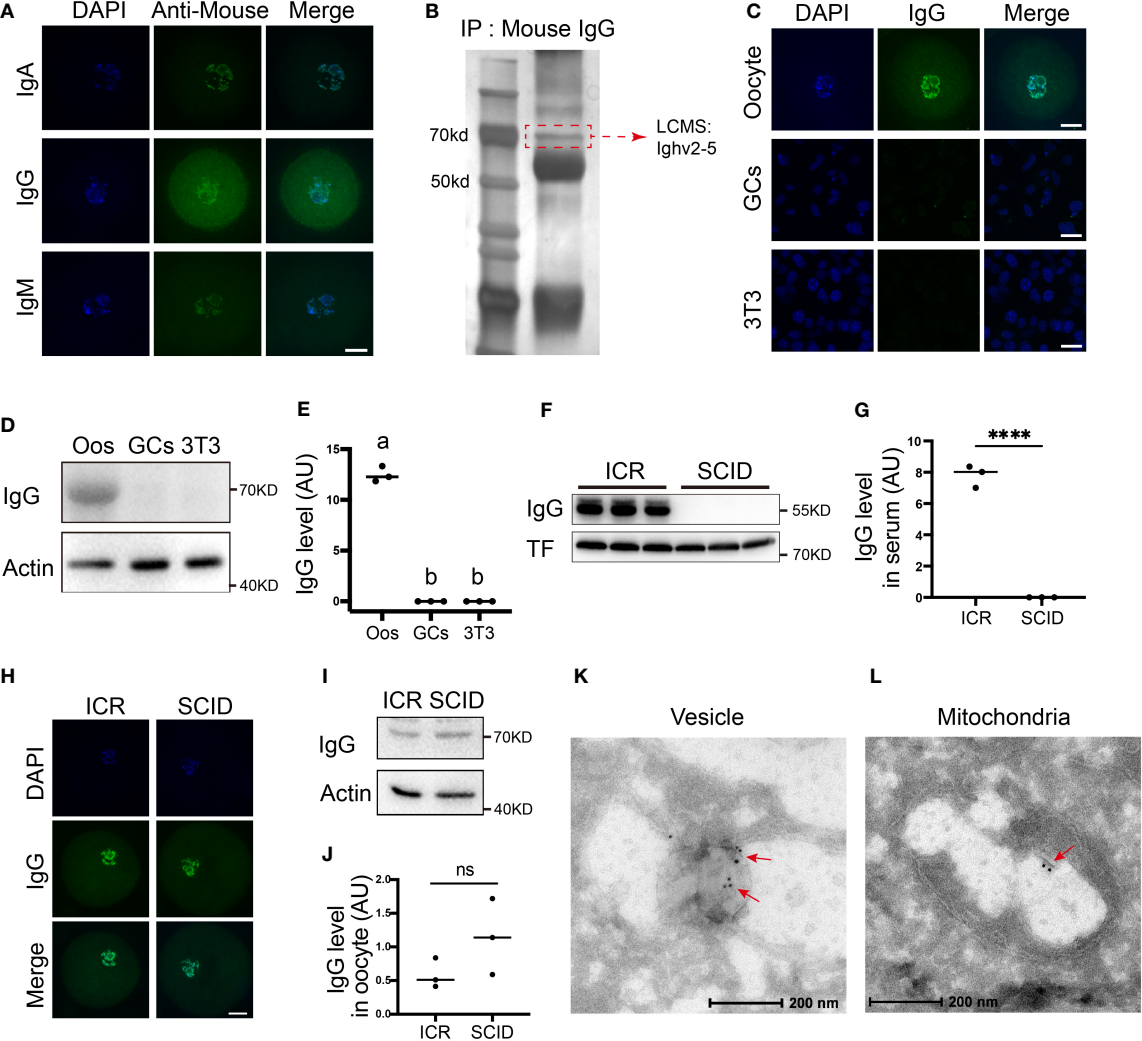
Immunoglobulins are present in oocytes **(A)** Immunofluorescence shows that different types of immunoglobulins, including immunoglobulin A (IgA), immunoglobulin G (IgG), and immunoglobulin G (IgM), are all present in mouse oocytes. **(B)** Immunoprecipitation with anti-mouse IgG, SDS-PAGE, silver staining, and LCMS show that the right-size band, which corresponds to the size of the IgG (dot-line square labeled) includes Ighv2-5 (Blue-highlighted in supplementary dataset 1). **(C)** Comparative immunofluorescence shows that IgG is rich in the oocytes but is rarely seen in granular cells (GCs) or NTH3T3 (3T3) cells. **(D, E)** Comparative Western blot and quantification show that IgG is rich in the oocytes but is not detectable in granular cells (GCs) or NTH3T3 (3T3) cells. **(F-J)** Immunofluorescence and Western blot show that IgG is rich in the serum of the control mice but completely absent in the serum of the immunodeficient SCID (server combined immune-deficiency) mice **(F, G)** however, IgG levels in the control ICR oocytes and SCID oocytes are quite similar **(H-J)**. K and L. Immuno-EM showed that IgG is present within diverse cytoplasmic organelles such as vesicle **(K)** and mitochondria **(L)**. Actin or transferrin (TF) was used as a loading control. Scale bars in other panels, 20 μm Scale bars in K and L, 200 μm. **** indicates p < 0.0001. ns, not significant.

We firstly examined the presence of IgG, IgA, and IgM in the oocytes through immunostaining and western blot. We clearly saw the presence of these immunoglobulins within oocytes (**Figure 1A**). Immunoprecipitation using the IgG antibody within oocyte lysate, SDS-PAGE, silver staining, and LCMS successfully identified IgG fragments (**Figure 1B**, blue-highlighted line in suppl. dataset 1). Because IgG is the major type of immunoglobulin, we focused on IgG. However, no IgG was seen in granular cells or somatic NIH3T3 cells (**Figures 1C–E**). Finally, we examined IgG levels in immunoglobulin-deficient SCID (severe combined immune-deficiency) mice and did not detect any IgG within their serum (**Figures 1F, G**); however, SCID oocytes had a level of IgG similar to that of control oocytes (**Figures 1H–J**). Finally, immuno-EM further verified the presence of IgG within diverse cytoplasmic organelles such as the vesicles (**Figure 1K**) and mitochondria (**Figure 1L**). These results suggest that immunoglobulin exists in oocytes independently of the overall immune system.

**Figure 2:**
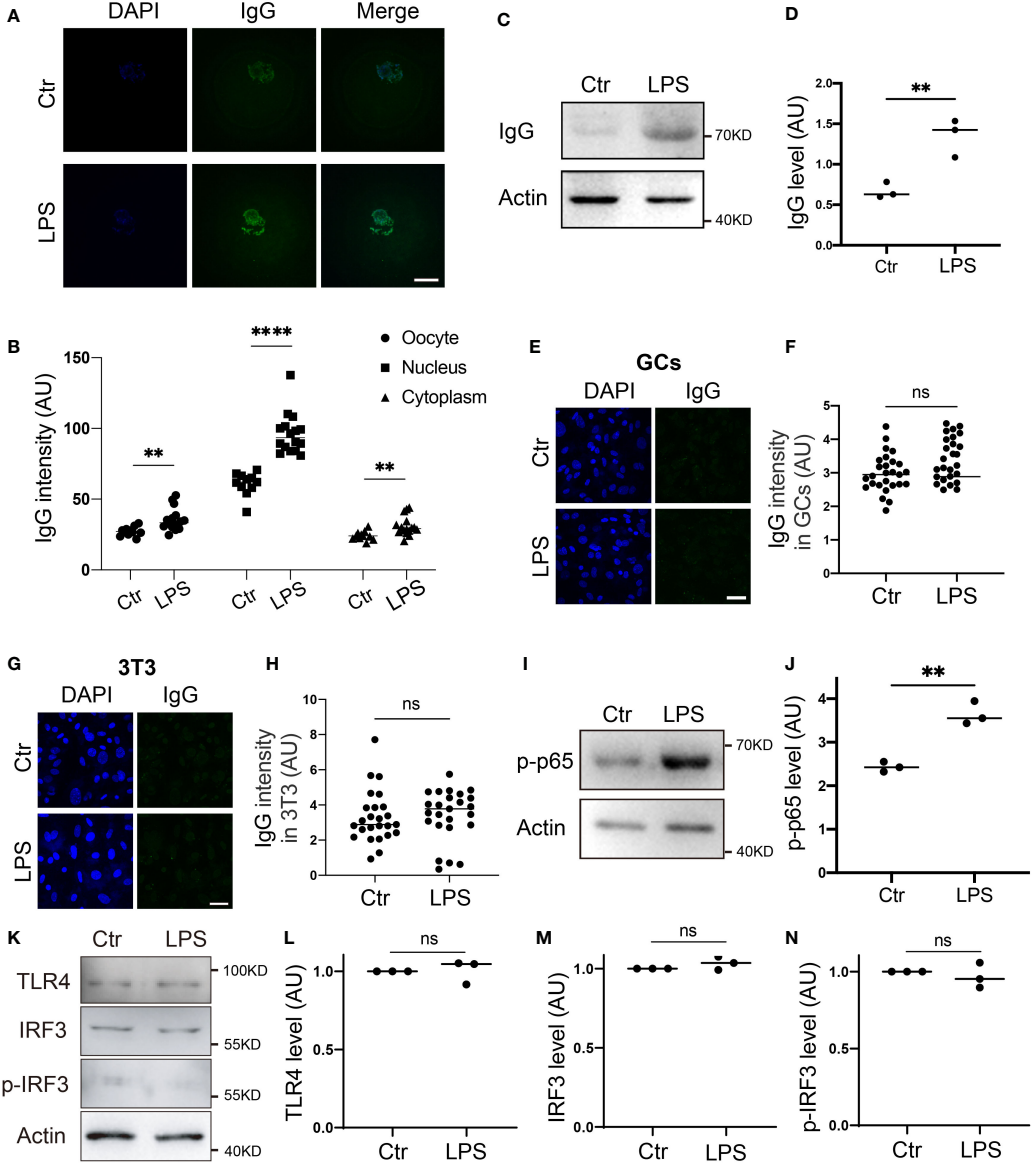
IgG protein increased in response to environmental LPS **(A, B)** Immunofluorescence shows that IgG increased more than one-fold in response to LPS, and nuclear IgG increased more significantly. **(C, D)** Western blot and quantification show that IgG protein level increased more than one-fold in response to LPS (Lipopolysaccharide) supplemented in the IVM (*in vitro* maturation) medium. **(E-H)** Immunofluorescence shows that IgG intensity is very low in control granular cells (GCs), **(E, F)** or NIH3T3 (3T3s), **(G, H)** and did not increase at all on exposure to LPS stimuli. **(I, J)** Western blot and quantification show that p-NFKB (p-p65) significantly increased in response to LPS. **(K-N)**. Western blot and quantification show that TLR4, IRF3, and p- IRF3 didn’t change at all in response to LPS. Actin was used as a loading control. Scale bars, 20 μm. ** indicates p < 0.01. **** indicates p < 0.0001. ns, not significant.

Generally, IgG levels increase in B lymphocytes in response to pathogens. We examined whether this could also occur in oocytes. We first used LPS, a common pathogen from gram-negative bacteria, to treat oocytes. Immunostaining and blotting both showed that the IgG level was significantly raised in the LPS-treated oocytes (**Figures 2A–D**), whereas neither the LPS-treated granular cells (GCs, **Figures 2E, F**) nor the NIH3T3 (3T3, **Figures 2G, H**) cells showed any increment in IgG levels. Moreover, we examined two key downstream effectors, p-NFKB (p-p65) and p-IRF3, which respond to extrinsic LPS *via* membrane CR2 (assisted by Complement) (16, 17) and TLR4 (18, 19) respectively, and found that p-p65 significantly increased upon extrinsic LPS stimuli (**Figures 2I, J**). Meanwhile, p-IRF3 levels did not change at all (**Figures 2K–N**).

These results suggest that immunoglobulins are present in mouse oocytes independently of the overall immune system, and that oocytes can rearrange IgG production mainly through LPS plus Complement → CR2 → p-p65 in response to environmental stimuli.

### Membrane receptor CR2 mediates IgG production in response to environmental LPS

In lymphocytes, besides through TLRs (Toll-like receptor) (20–22), the transduction of extrinsic immunogenic signals in the cells can also be mediated through several other types of receptors, such as CD46 (CD46 antigen, complement regulatory protein) (23), FCGR1A (Fc fragment of IgG receptor Ia) (24), and CR2 (complement receptor 2) (25). Interestingly, these receptors are present within the oocyte membrane (13). Therefore, we wanted to know which receptor mediated IgG production in response to immunogenic extrinsic LPS.

**Figure 3:**
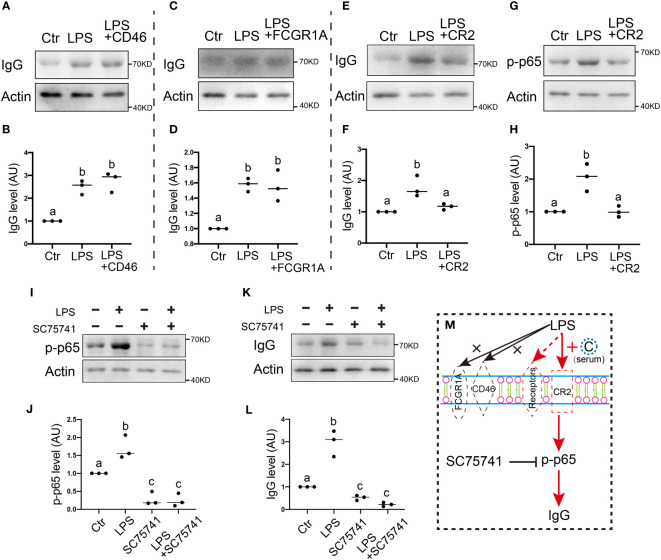
. Membrane receptor CR2 mediates IgG production in response to environmental LPS **(A, B)** Western blot and quantification show that on exposure to LPS stimuli, IgG levels significantly increased about one-fold, and CD46 (CD46 antigen, complement regulatory protein) blockage with the anti-CD46 antibody had no impact on the LPS-induced IgG increment. **(C, D)** Western blot and quantification show that FCGR1A (Fc fragment of IgG receptor Ia) blockage with anti-FCGR1A antibody had no impact on the LPS-induced IgG increment. **(E-H)** Western blot and quantification show that CR2 (complement receptor 2) blockage with anti-CR2 antibody brought the LPS-induced IgG increment back to the control level **(E, F)** CR2 blockage also brought LPS-induced p-p65 increment back to the control level **(G, H)**. **(I-L)** Western blot and quantification show that on exposure to LPS stimuli, both p-p65 and IgG level significantly increased about one-fold, and the NFKB inhibitor SC75741 brought the LPS-induced p-p65 or IgG increment back to the control level. **(M)** Model hypothesis for A-L. LPS-induced increment of p-p65→IgG is mediated by membrane receptor CR2, not CD46 or FCGR1A. Actin was used as a loading control. Difference lower-case letters indicate significant difference between two groups.

We found that, on exposure to LPS stimuli, the IgG level significantly increased one-fold, while CD46 blockage with the anti-CD46 antibody had no impact on the IgG increment on exposure to LPS stimuli (**Figures 3A, B**). Similarly, FCGR1A blockage with the anti-FCGR1A antibody had no impact on the IgG increment on exposure to LPS stimuli (**Figures 3C, D**). In contrast, CR2 blockage with the anti-CR2 antibody largely counteracted the IgG increment on exposure to LPS stimuli (**Figures 3E, F**).

The NFKB pathway played an essential role in regulating IgG production inside the lymphocytes (26, 27), Next, we wanted to know whether this was also true for oocytes. We found that CR2 blockage with the anti-CR2 antibody also largely counteracted the p-p65 increment on exposure to LPS stimuli (**Figures 3G, H**), while inhibition of the NFKB pathway with a specific inhibitor SC75741 (**Figures 3I, J**) largely counteracted the IgG increment on exposure to LPS stimuli (**Figures 3K, L**).

These results suggest that membrane CR2 is essential for IgG increment on exposure to LPS stimuli, whereas NFKB is also essential inside the oocytes, probably because it receives the activation upstream signal from CR2 (**Figure 3M, N**).

### IgG transcription increased and was regulated by H3K27ac on exposure to CSF1 or LPS stimuli

Generally, from the perspective of *IgG* genes, B cells are distinct from each other, highly-variable regions, comprising hundreds of V, D, and J genes, undergo pre-setup recombination for each specific potential immunogen. On exposure to a certain immunogen stimulation, a B cell with a corresponding recombined VDJ type is awakened and clones itself into a colony of B cells with the same VDJ type. Oocytes contain IgG even in the absence of environmental immunogen stimulation, and they increase IgG production on exposure to stimulation. Therefore, we are interested to know whether oocytes are distinct from each other in terms of IgG VDJ regions without immunogen stimulation, and whether the VDJ region undergoes recombination again on exposure to stimulation.

**Figure 4:**
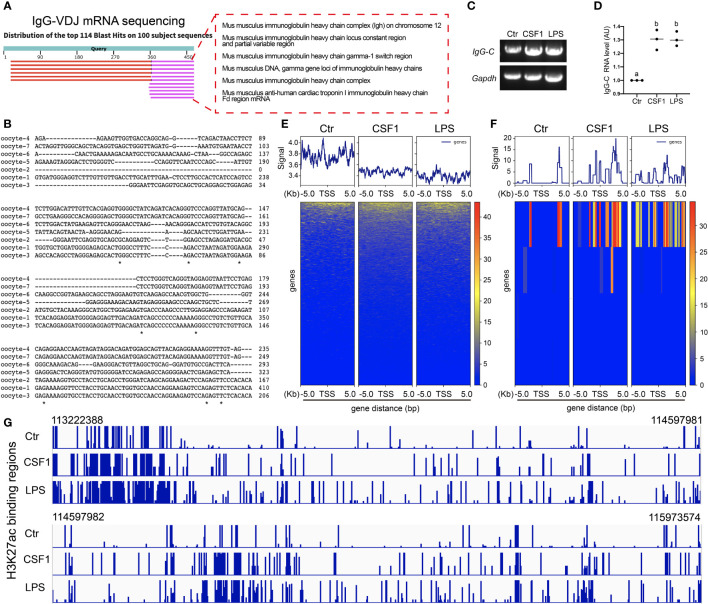
IgG transcription increased and was regulated by H3K27ac **(A)** Amplification of IgG-VDJ region, cloning and sequencing shows that IgG-VDJ mRNA is present in the oocyte. **(B)** Parallel comparison of IgG-VDJ mRNA sequences from **(A)** shows that IgG-VDJ mRNAs are highly distinct between different oocytes. **(C, D)** Amplification of IgG constant (IgG-C) region showed that IgG-C mRNA levels significantly increased on exposure to either LPS or CSF1 stimuli. **(E, F)** H3K27ac chip-seq showed that on exposure to either LPS or CSF1 stimuli, H3K27ac decreased its binding to the regulating sequences of all genes however, its binding to the regulating sequences of the IgG gene significantly increased. **(G)** Magnified H3K27ac-binding regions in IgG regulating sequences showed that upon both CSF1 and LPS, H3K27ac increased its binding. *Gapdh* was used as a loading control. Difference lower-case letters indicate significant difference between two groups.

First, we found that *IgG* mRNA, as indicated by the PCR product of the *IgG* VDJ region, existed in oocytes (**Figure 4A**) without immunogen stimulation; moreover, sequencing showed that the VDJ region was distinct between randomly-examined individual oocytes (**Figure 4B**). Next, we found that gross *IgG*-constant mRNA increased on exposure to CSF1 or LPS stimuli (**Figures 4C, D**).

Post-translation modified histones—for example, H3K4me^3^ and H3K27ac (28)—have been confirmed to bind to the upstream regulating sequences to promote IgG transcription. We wanted to see whether the histones regulate IgG transcription, and if so, which histones. CHIP-seq showed that on exposure to either LPS or CSF1 stimuli, H3K27ac decreased its binding to the regulating sequences of all genes (**Figures 4E–G**); however, its binding to the regulating sequences of the *IgG* gene significantly increased (**Figures 4F, G**). In contrast, H3K4me^3^ did not show significantly increased binding on exposure to LPS or CSF1 stimuli (**Suppl. Figures 1A–C**).

### IgG functions to eliminate ROS production in response to environmental stimuli

It appeared that oocytes perform like immune cells to adjust IgG production. Next, we wanted to see whether IgG is functionally important for the maintenance of quality and survival of the oocyte itself.

**Figure 5:**
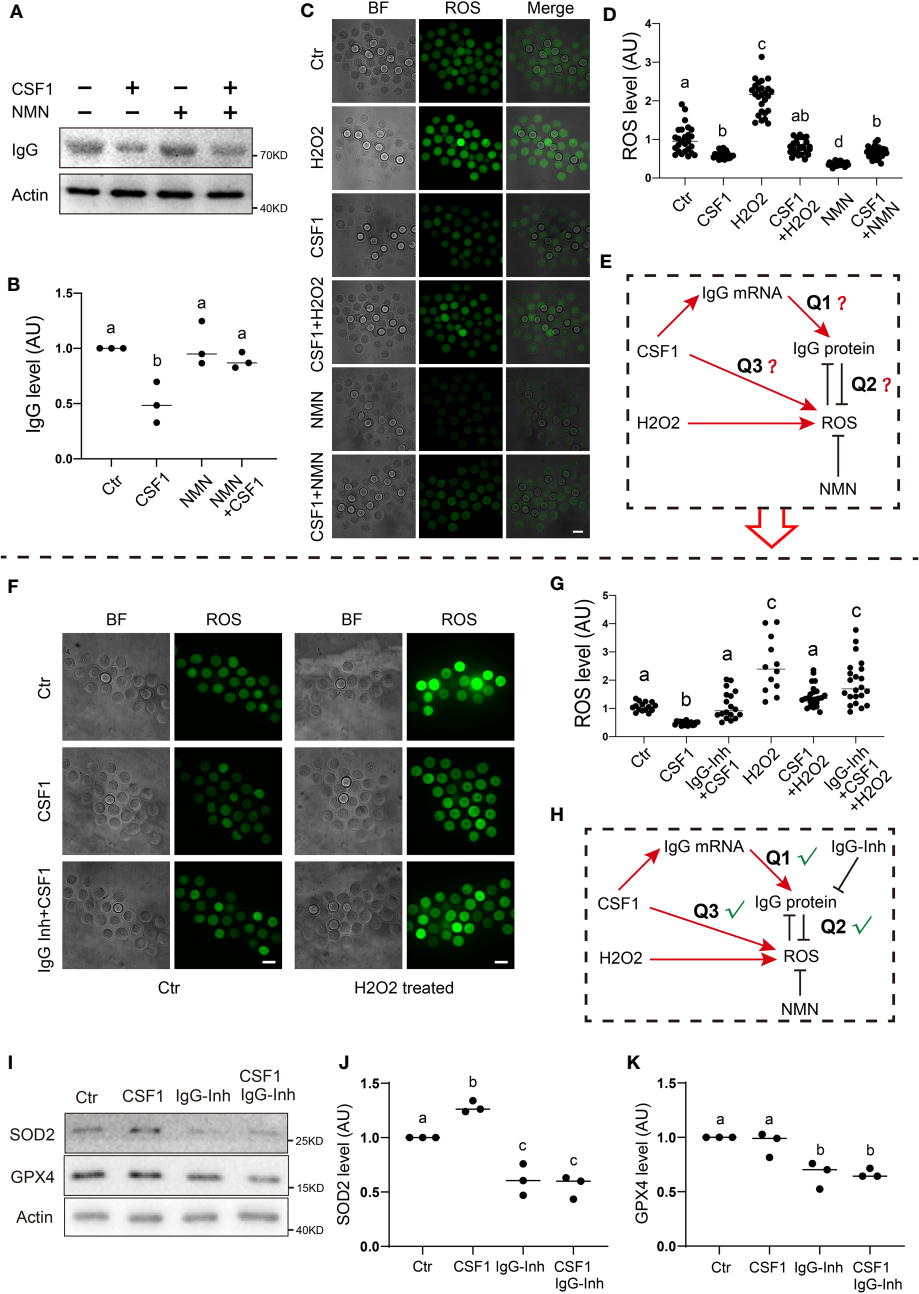
IgG functions to eliminate ROS production in response to CSF1 stimuli **(A, B)** Western blot and quantification show that on exposure to CSF1 stimuli, IgG levels significantly decreased about one-fold. NMN supplement significantly raised the IgG level close to the control level. **(C, D)** ROS live fluorescence shows that on exposure to CSF1 stimuli, ROS significantly decreased by about 40.1% on exposure to NMN supplement, ROS significantly decreased over one-fold on exposure to H2O2 stimuli, ROS significantly increased by more than one-fold. CSF1 co-supplement with NMN raised the ROS level in NMN-treated oocytes to close to the control level CSF1 co-supplement with H2O2 reduced the ROS level in H2O2-treated oocytes to near the control level. **(E)** Model hypothesis for **(A–D)** CSF1-induced IgG increment might be used to eliminate ROS production on exposure to either CSF1 or H2O2 treatment. **(F, G)** ROS live fluorescence shows that on exposure to CSF1 stimuli, ROS significantly decreased by about 53.7% while IgG co-inhibition significantly raised the ROS level close to the control level. On exposure to H2O2 stimuli, ROS significantly increased by more than one-fold the CSF1 co-supplement reduced the ROS level in H2O2-treated oocytes to close to the control, whereas IgG inhibition+CSF1+H2O2 significantly increased the ROS level compared with the CSF1+H2O2 group. **(H)** Model hypothesis for A-G: CSF1 treatment could induce both IgG and ROS increment increased IgG can be used to counteract the increment of ROS on exposure to CSF1 or H2O2 stimuli. **(I-K)** Western blot and quantification show that SOD2 significantly increased in response to CSF1, while decreased upon IgG inhibition or CSF1 treatment plus IgG inhibition GPX4 level was not affected by CSF1 treatment but decreased upon IgG inhibition. Actin was used as a loading control. Scale bars, 20 μm. Difference lower-case letters indicate significant difference between two groups.

We first examined the relationship between the IgG level and the ROS level, which is essential for cell quality (29). We found that, on exposure to CSF1 stimuli, the IgG level significantly decreased approximately one-fold (**Figures 5A, B**), whereas the ROS level also decreased (**Figures 5C, D**). In contrast, the H2O2 treatment significantly elevated the ROS level, whereas the CSF1 treatment pulled the H2O2-induced ROS level back to the control level (**Figures 5C, D**). Next, co-treating CSF1-stimulated oocytes with NMN, a well-known ROS remover, pulled the CSF1-induced IgG level back to the control level (**Figures 5A, B**), and CSF1/NMN co-treated oocytes had significantly higher ROS levels than NMN-treated oocytes. These phenomena led us to presume that CSF1 stimulation could increase both ROS and IgG protein level, whereas increased IgG acts as an endogenous anti-oxidant to counteract excessive toxic ROS (**Figure 5E**). In further support, IgG inhibition counteracted the CSF1-induced decrease in ROS, whereas IgG inhibition counteracted the ROS decrement caused by co-treating H2O2-challeging oocytes with CSF1 (**Figures 5F, G**).

Furthermore, we found that the level of two free-radical-detoxifying enzymes, SOD2 (Superoxide dismutase 2) and GPX4 (Glutathione peroxidase 4), also significantly decreased upon IgG inhibition in the presence or absence of CSF1 (**Figures 5I–K**).

In summary, all the evidence supported that IgG could be utilized to reduce the superfluous detrimental ROS induced by environmental stimuli, such as CSF1 challenge (**Figure 5H**).

### IgG functions to protect mitochondria integrity in response to virus ssRNA stimuli

**Figure 6:**
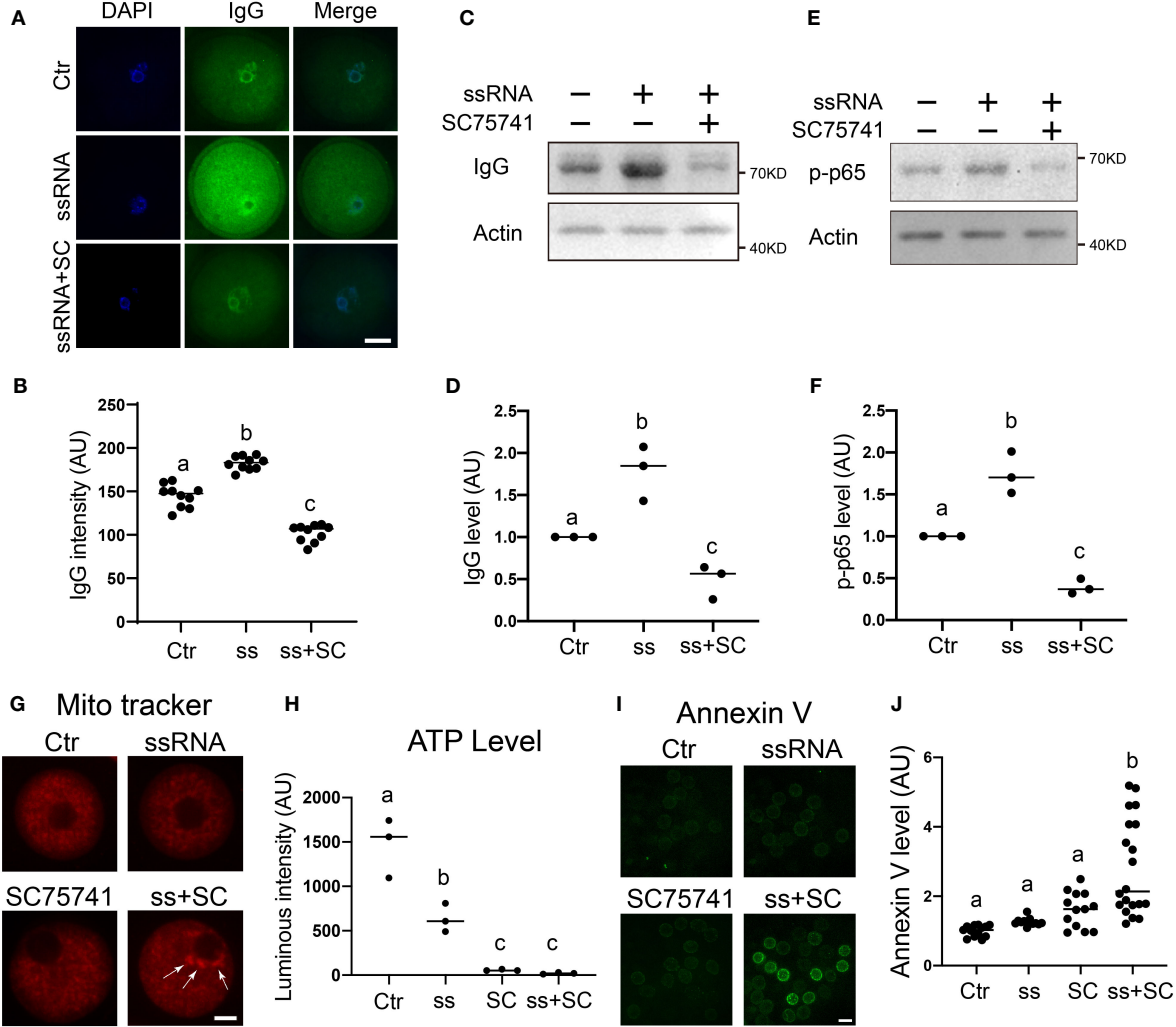
IgG functions to protect mitochondria integrity in response to virus ssRNA stimuli **(A, B)** Immunofluorescence shows that IgG intensity significantly increased in virus ssRNA-injected oocytes. DNA in blue, IgG in green. **(C–F)** Western blot and quantification show that upon virus ssRNA injection, the IgG level significantly increased, whereas the NFKB inhibitor SC75741 significantly brought the ssRNA-induced IgG increment back to the control level. The p-p65 level also significantly increased upon ssRNA injection, whereas the SC75741 significantly brought the ssRNA-induced p-p65 increment back to the control level. **(G, H)** Mitochondria live fluorescence showed that both ssRNA injection and NFKB inhibition caused increased mitochondria aggregation, accordingly ATP level significantly decreased upon either treatment. While ssRNA injection combined with NFKB inhibition further exacerbate mitochondria aggregation, accordingly ATP level further decreased. **(I, J)** Live fluorescence showed that early apoptotic level indicated by Annexin V significantly increased upon ssRNA injection combined with NFKB inhibition. Actin was used as a loading control. Scale bars, 20 μm. Difference lower-case letters indicate significant difference between two groups.

Virus ssRNA (single-strand RNA) has been shown to elicit the inflammation and immune response of host cells (30). Next, we examined how IgG functions in response to virus ssRNA. We used a mixture of two GU-rich ssRNA to mimic virus RNA as reported (31). We found that ssRNA injection significantly increased IgG level (**Figures 6A–D**), while inhibition of the NFKB pathway pulled ssRNA-elevated IgG back toward the control level (**Figures 6C, D**). Furthermore, ssRNA injection significantly increased the p-p65 level, which was also able to be pulled back to control by SC75741 (**Figures 6E, F**).

Next, we found that both ssRNA injection and NFKB inhibition caused increased mitochondrial aggregation; accordingly, ATP level significantly decreased upon either treatment. Furthermore, ssRNA injection combined with NFKB inhibition further exacerbated mitochondrial aggregation, consequently decreasing ATP levels further (**Figures 6G, H**).

Finally, we found that the early apoptotic level, as indicated by Annexin V, significantly increased upon ssRNA injection combined with NFKB inhibition (**Figures 6I, J**).

These results indicated that virus ssRNA-induced, NFKB-mediated IgG increment could protect mitochondrial integrity from ssRNA-induced inflammation.

### IgG functions to promote oocyte survival in response to *E. coli* stimuli

Although oocytes are normally embedded within the ovaries and protected from environmental infection, they could be impacted by severe physical infection such as severe sepsis (14), severe salpingitis (15), or a cytokine storm during virus infection (15). Therefore, we next wanted to determine whether IgG is active during infection, and how it functions.

**Figure 7:**
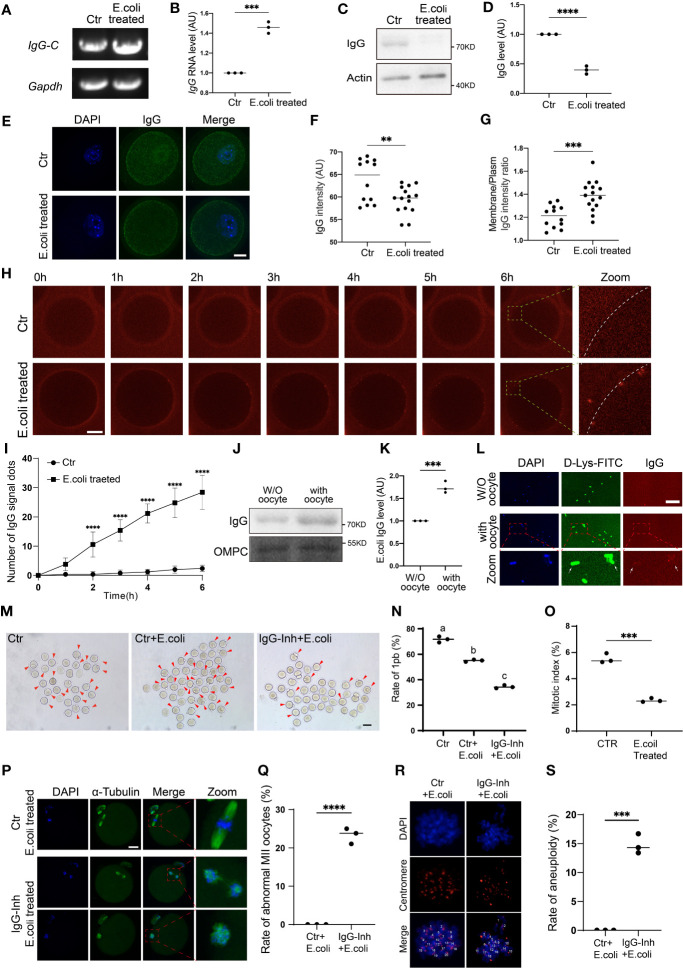
IgG functions to protect oocyte survival in response to E. coli stimuli **(A–D)**. **(E)** coli was added to the oocyte IVM medium. RT-PCR and quantification show that IgG-constant (IgG-C) mRNA level significantly increased on exposure to E. coli stimuli **(A, B)**, whereas Western blot shows that IgG protein level significantly decreased **(C, D)**. **(E–G)** Immunofluorescence shows that on exposure to **(E)** coli stimuli, IgG intensity within oocytes significantly decreased, however, the membrane/cytoplasm IgG ratio significantly increased. **(H, I)** Live image of fluorescence dye (Cy2)-labeled IgG and quantification show that E. coli supplemented into the oocyte IVM medium caused increased IgG aggregates on the oocyte membrane. **(J, K)** Western blot shows that after **(E)** coli is supplemented into the oocyte IVM medium, the IgG protein level significantly increased in E. coli pellets. **(L)** Immunofluorescence shows that E. coli supplemented into the oocyte IVM medium caused significantly increased IgG staining on E. coli. A region in oocyte-challenging E. coli was magnified and is shown below. DNA in blue, E. coli (D-Lys-FITC labeled) in green, IgG in red. **(M–O)** IVM and quantification show that the mitosis and meiosis decrement extent of E. coli-challenged NIH3T3 (from 5.52% to 2.35%, O) was much larger than that of E. coli-challenged oocytes (from 71.65% to 55%, N). However, IgG inhibition further decreased the oocyte maturation rate from 55% to 34.4% **(N)**. P. Side-by-side comparison of E-coli-treated NIH3T3 cells and oocytes showed that the decrement extent of the mitotic index was much larger than that of the oocyte maturation rate. **(P, Q)** Immunofluorescence and quantification showed that IgG inhibition caused a significantly increased percentage of maturated oocytes with an abnormal chromosome alignment and spindle structure. **(R, S)** Chromosome spread, immunofluorescence and quantification of aneuploidy showed that IgG inhibition caused a significantly increased percentage of MII oocytes with aneuploidy. *Gapdh*, Actin or OMPC (Escherichia coli Outer Membrane Pore Protein C) was used as a loading control. Scale bars, 20 μm. ** indicates p < 0.01, *** indicates p < 0.001. **** indicates p < 0.0001. Difference lower-case letters indicate significant difference between two groups.

We first found that IgG-constant mRNA significantly increased on exposure to *Escherichia coli* stimuli (**Figures 7A, B**), whereas the western blot showed that IgG protein levels significantly decreased (**Figures 7C, D**). Then, immunofluorescence also showed that, on exposure to *E. coli* stimuli, IgG intensity within oocytes significantly decreased (**Figures 7E, F**); however, the membrane/cytoplasm IgG ratio significantly increased (**Figures 7E, G**). Next, we injected cy2-conjugated donkey anti-mouse IgG into the oocytes and tracked the dynamics of IgG. Live imaging showed that after two hours of *E. coli* stimuli, large IgG aggregates gradually increased across the whole membrane, from the inner side to outer side (**Figures 7H, I**), indicating that on exposure to *E. coli* stimuli, IgG could aggregate onto the membrane and then be secreted out of the oocytes. However, we did not see the bright IgG dots at the outside of the zona pellucida (ZP), probably due to the quick disassembly of the IgG aggregates once they were secreted out of the ZP. However, oocyte-treating *E. coli* showed significantly increased IgG (**Figures 7J–L**), suggesting that the excreted IgG could encompass and precipitate *E. coli*.

Next, we examined how the increased IgG would be beneficial for *E. coli*-challenged oocytes. *In-vitro* maturation (IVM) results showed that *E. coli*- challenged oocytes had a 23.23% decreased maturation rate compared with control oocytes (**Figures 7M, N**, rate of 1pb, first polar body, *E. coli*-challenged vs. control, 71.65% vs. 55%); In contrast, the *E. coli*-challenging of NIH3T3 cells reduced their mitotic indexes from 5.52% to 2.35% (**Figure 7O**). Apparently, the extent of maturation decrement of oocytes was much smaller than that of NIH3T3 cells upon *E. coli* challenge (**Figures 7N, O**). However, oocytes co-treated with IgG-inhibition and *E. coli* had more than a one-fold decreased maturation rate (**Figures 7M, N**, rate of 1pb, *E. coli* plus IgG inhibition vs. control, 71.65% vs. 34%).

Finally, although their maturation rate was decreased on exposure to *E. coli* treatment, the maturated MII oocytes had normal chromosome alignment; however, IgG inhibition together with *E. coli* treatment significantly increased the percentage of MII oocytes with abnormal chromosome alignment (**Figures 7P, Q**) and aneuploidy (**Figures 7R, S**).

These results suggested that oocytes could increase IgG production to survive over bacterial infection.

## Discussion

In the present study, we not only found that immunoglobulins exist within mouse oocytes, but also verified that IgG was important for the self-protection and quality maintenance of oocytes in response to environmental stimuli. Furthermore, we also uncovered how immunoglobulin level was regulated.

Immunoglobulins are key humoral immune molecules produced and secreted by B lymphocytes at various stages of differentiation. However, several other types of cells, such as sperm (8), islet cells (9), hepatocytes (10), umbilical cord endothelial cells (11) and eye cells (12) have also been reported to contain immunoglobulins. However, these studies only showed some of the functional relevance of IgG in these cells. In the present study, through a logical experimental series design, we uncovered at least three aspects of the important biological function of oocyte IgG, which support that oocyte IgG could exert similar immune function to lymphocytes. Moreover, we also found both similarities and differences in the ways oocyte and lymphocyte IgG function.

In terms of IgG structure, rearrangement of the VDJ region could occur even before stimuli in both oocytes and lymphocytes, and the VDJ regions of individual cells were distinct from each other. However, in the western blot test, the molecular size of the oocyte IgG heavy chain was about 70 KDa, whereas the lymphocyte IgG heavy chain was about 55 KDa; thus, oocyte IgG is more like IgY. Moreover, the oocyte IgG level remained unchanged in the SCID mice, suggesting that oocyte IgG was independent of the overall immune system, and oocyte IgG could protect itself even under conditions of systemic immunodeficiency, which agree with the observation that HIV patients can have normal fertilization, early embryo development, and live birth (32, 33).

For the upstream signal pathway that regulates IgG level, NFKB is the essential mediator that receives signals from the membrane and enters the nucleus to regulate *IgG* transcription for both oocytes and lymphocytes. However, oocytes appeared to receive signals from external stimuli mainly through CR2 instead of CD46 or FCGR1A, while all of these receptors sense outer stimuli in lymphocytes.

For the epigenetic regulation of the *IgG* gene, modified histone can regulate *IgG* transcription in both oocytes and lymphocytes (28). However, in oocytes, H3K27ac showed positive binding, while H3K4me^3^ showed negative binding onto *IgG* regulating sequences on exposure to both CSF1 and LPS stimuli, whereas in lymphocytes, H3K4me3 showed positive binding onto *IgG* regulating sequences (28).

Overall, in the present study, we found that the oocyte could rearrange IgG production to protect itself from adverse environmental stimuli. Although the mechanism is partially different from that of lymphocytes, oocytes behave like immune cells. However, because the oocyte cannot reproduce itself, the IgG can only be used for self-protection. Further investigation is required to uncover more detailed mechanisms and the functional relevance of oocyte IgG.

## Materials and methods

### Mice

Animal experimental procedures in our study were all approved by the Animal Ethics Committee of Nanjing Medical University (NJMU) (approval no. IACUC-1809011), and all mice were housed in the Animal Core Facility (ACF) of NJMU under standard specific pathogen free (SPF) conditions of ACF.

Three-week-old SCID (Severe Combined Immunodeficient) mice were bought from Beijing Vital River Laboratory Animal Technology Co., Ltd. (Beijing, China) and housed in the ACF.

Three-week-old control CD1/ICR mice were bought from ACF.

### Antibodies

Primary antibodies: Anti Beta Actin mAb (Cat#:S6007M, Bioworld, Dublin, OH, USA); Rabbit anti OMPC (Cat#: bs20213R, Bioss Antibodies, Beijin, China); Acetyl-Histone H3(K27)(D5E4) XP(R) rabbit mAb (Cat#: 8173s, Cell Signaling Technology, Danvers, MA, USA); Histone H3 (Tri-Methyl K4) pAb (Cat#: BS7232, Bioworld, Dublin, OH, USA); CR2 rabbit pAb (Cat#: A8407, ABclonal, Wuhan, Hubei, China); CD46 rabbit pAb(Cat#: A1653, ABclonal, Wuhan, Hubei, China); FCGR1A pAb (Cat#: BS6238, Bioworld, Dublin, OH, USA); Phospho-Histone H3(Ser 10) Mouse McAb (Cat#: 66863-1-Ig; Proteintech, Rosemont, IL, USA); Tubulin α (G436) mAb (Cat#: BS1699M; Bioworld, Dublin, OH, USA); Anti-RELA (Phospho-Ser536) rabbit polyclonal antibody (Cat#: D155006; BBI, Shanghai, China), AffiniPure Goat anti-Mouse IgG, light chain specific (Jackson ImmunoResearch Laboratory, Cat#: 115-005-174, West Grove, PA, USA); SOD2 Rabbit Recombinant mAb (Cat#: A5377; Houston, TX, USA); GPX4 Rabbit Recombinant mAb (Cat#: A5569; Houston, TX, USA); IRF3 Rabbit Recombinant mAb (Cat#: A5373; Houston, TX, USA); TLR4 rabbit pAb (Cat#: A11226, ABclonal, Wuhan, Hubei, China); Phospho-IRF3 (Ser396) Rabbit Polyclonal Antibody (Cat#: A8407, Beyotime, Tangshan, Hebei, China).

Secondary antibodies: Horseradish peroxidase (HRP)-conjugated rabbit anti-goat IgG and HRP-conjugated goat anti-mouse IgG were purchased from Jackson ImmunoResearch Laboratory (West Grove, PA, USA). Goat anti-mouse IgA-FITC (Bersee, Cat#: BFR522, Beijing, China); Goat anti-mouse IgM-FITC (Bersee, Cat#: BFR523, Beijing, China); Cy2-conjugated donkey anti-mouse IgG (Code: 715-225-150), Cy3-conjugated donkey anti-mouse IgG (Code: 715-165-150), all purchased from Jackson ImmunoResearch Laboratory (West Grove, PA, USA).

### Oocyte collection and *in vitro* culture

Fully-grown GV oocytes were collected from three-week-old female control or SCID mice. Oocytes were released by puncturing follicles with a sterile syringe needle in a MEM+ medium (0.01 mM EDTA, 0.23 mM Na-pyruvate, 0.2 mM penicillin/streptomycin and 3 mg/ml BSA in MEM). After the cumulus cells from the cumulus-oocyte complexes (COC) were washed off, cumulus-free oocytes were cultured in 100 µl mini-drops of MEM+ containing 10% fetal bovine serum (FBS) (Thermo Fisher) covered with mineral oil at 37.0°C in an incubator with 5% O_2_, 5% CO_2_, and a humidified atmosphere.

### Cell culture and mitotic index assay

NIH3T3 cells were from ATCC (Cat No.: CRL-1658) and sold by Procell Life Science and Technology Co. (Wuhan, Hubei, China). The cells were cultured in DMEM with 10% FBS. For mitotic index assay, NIH3T3 cells were fixed and immunostained for tubulin and p-H3, the cells with strong p-H3 staining on the chromosomes are mitotic cells. For each sample, at least 1000 cells were counted. Mitotic index is the number of mitotic cells divided by the number of total counted cells.

### Immunofluorescence staining of oocytes and image taking

Two distinct protocals were used for the permeation and fixation of oocytes. The blocking, primary antibody incubation, secondary antibody incubation and oocyte mounting procedures after permeation and fixation are the same for these two protocals.

Permeation and fixation:

1). For experiments in **Figures 7E–G**, we wanted to maintain IgG signals furthest on the membrane. After three times of quick wash in PBS/0.05% PVP (polyvinylpyrrolidone), oocytes were first fixed in 3.7% FPA in PHEM for 30 minutes at room temperature and washed with PBS/PVP three times at 10 minutes each. Then oocytes were permeated in 1% Triton X-100/PHEM (60 mM PIPES, 25 mM Hepes, pH 6.9, 10 mM EGTA, 8 mM MgSO_4_) for 10 minutes.
2). For all other experiments except **Figures 7E–G**, we wanted to get best quality of cytoplasmic signal. After three times of quick wash in PBS/PVP, oocytes were first permeabilized with 0.5% Triton X-100/PHEM (60 mM PIPES, 25 mM Hepes, pH 6.9, 10 mM EGTA, 8 mM MgSO_4_) for 5 minutes. After washed with PBS/PVP three times at 10 minutes each, oocytes were fixed in 3.7% FPA in PHEM for 20 minutes at room temperature.

Blocking, primary antibody incubation, secondary antibody incubation and oocyte mounting:

After being washed with PBS/0.05% PVP (polyvinylpyrrolidone) three times at 10 minutes each, oocytes were blocked in blocking buffer (100 mM glycine and 1% BSA in PBS) for one hour at room temperature. Primary antibodies were then diluted in blocking buffer and oocytes were incubated in it overnight at 4.0°C. Fluorescent secondary antibodies (Jackson ImmunoResearch Laboratories) were used at 7.5 μg/ml. DNA was stained with 0.3 μg/ml Hoechst 33258. Next, buffers were slowly and gently removed from around the oocytes, which immobilized the oocytes on the slide; then a drop (about 5-10 μl) of anti-fade solution (0.25% n-propyl gallate and 90% glycerol in PBS) was mounted onto the oocytes and oocytes were covered by a coverglass. To avoid the deformation of the oocytes, a double-stick tap was pre-placed between the slides and coverslips. Specimens were imaged with IQ2 on an Andor Revolution spinning-disk confocal system (Andor Technology PLC, Belfast, UK) mounted on an inverted TiE microscope (Nikon, Japan) with a 60 ×, 1.4 NA objective and captured with cold CCD camera (Andor). Most images are displayed as maximum intensity projections of the captured z stacks.

### Immunoprecipitation, coomassie staining and mass-spec

2.5 μg HRP-conjugated goat anti-mouse IgG antibody was first coupled to 30 μl protein-A/G beads (Yeasen, Shanghai, China) for 4 hours at 4°C on a rotating wheel in 250 μl immunoprecipitate (IP) buffer (20 mM Tris-HCl, pH 8.0, 10 mM EDTA, 1 mM EGTA, 150 mM NaCl, 0.05% Triton X-100, 0.05% Nonidet P-40, 1 mM phenylmethylsulfonyl fluoride) with 1:100 protease inhibitor (Sigma) and 1:500 phosphatase inhibitor (Sigma). Meanwhile, 400 oocytes were lysed and ultrasonicated in 250 µl IP buffer and then pre-cleaned with 30 µl protein-A/G beads for 4 hours at 4°C. After that, protein A/G-coupled Rabbit IgG or specific antibody were incubated overnight at 4°C with pre-cleaned oocyte lysate supernatant. Finally, after being washed three times (10 minutes each with 250 µl IP buffer), the resulting beads with bound immunocomplexes were subjected to SDS-PAGE and coomassie staining. The gel band corresponding to the band from IgG blot was cut out and sent to Biotech-pack (Beijing, China) for mass spec identification of proteins.

### CHIP-seq

H3K27ac and H3K4me^3^ CHIP-seq were perform with Hyperactive *In-Situ* ChIP Library Prep Kit under Targets and Tagmentation (CUT&Tag) technology (Vazyme) according the manufacturer’s instruction. The kit using a hyperactive Tn5 transposase to precisely binds the DNA sequence near the target protein. In brief, lysate of 100 oocyte were incubated with ConA beads, then primary and secondary antibody were in turn incubated with oocyte lysate, next Hyperactive pG-Tn5/pA-Tn5 Transposon was incubated with the sample and tagmentation were performed. Finally DNA was extracted and library was prepared as instructed, and DNA library was sent to Novogene (Beijing, China) for sequencing.

### Virus SSRNA production and microinjection

Immunity-inducing GU-rich virus ssRNA (Single-strand RNA) sequences were according to reported (23). We selected two sequences: 5’-GACTTGAGCGAGCGCTTTTTT-3’ (ssRNA-6T) and 5’-GTCCGGGCAGGTCTACTTTTT-3’ (ssRNA-5T). SsRNA were produced and purified using the T7 Ribomax Express RNAi System (Promega) according to the manufacturer’s instructions, then aliquoted and stored at -80°C. The difference here is that the RNA is single-stranded from one transcription reaction. Primers for DNA templates are in suppl. table 2.

SsRNA was adjusted to 5uM with nuclease-free water, and about 10 pl of ssRNA was injected into GV oocytes with a programmed microinjector. 10 μM milrinone was included to prevent GVBD.

### LPS, CSF1, H2O2, NMN and SC75741 treatment

LPS (Cat#: L2880, Sigma, St. Louis, MO, USA) was added into oocyte IVM medium at a final conc. of 10 EU/ml for 16 hour; CSF1 (Cat#: HZ-1207, Proteintech, Rosemont, IL, USA) was added into oocyte IVM medium at a final conc. of 10 ng/ml for 16 hour; H2O2 (Cat#: 88597, Sigma, St. Louis, MO, USA) was added into oocyte IVM medium at a final conc. of 30 mM for 30 min; NMN (Cat#: S5259, Selleck Chemicals, Houston, TX, USA) was added into oocyte IVM medium at a final conc. of 1 mM for 16 hour; SC75741 (Cat#: S7273, Selleck Chemicals, Houston, TX, USA) was added into oocyte IVM medium at a final conc. of 0.1 μM for 16 hour.

### E-coli fluorescence labeling and treating oocytes with E-coli

FICT-d-Lys(Cat#: I0201,Bioluminor, XiaMen, Fujian, China)was added into LB medium at a final conc. of 0.1mM. 2.5 μl DH5α competent E.coli (Cat#: C502, Vazyme, Nanjing, Jiangsu, China) were added into 1 ml LB medium and the E.coli culture were grown at 37°C until OD600 reached 0.6. Then E.coli was collected and washed by PBS, then re-suspend 1ml PBS. Finally, 10 μl PBS/with Ecoli was added into 100 μl IVM medium with 50 oocytes during the whole IVM period. Except for maturation assay done at 16 hour of E.coli treatment, all other E.coli-treated oocyte experiment were done at 6 hour.

### Antibody inhibition

Antibodies for CR2, CD46 and FCG1R were added into oocyte IVM medium at a final conc. of 0.05 mg/ml to inhibit corresponding receptor on the oocyte membrane. 0.065 mg/ml antibody for IgG light chain was microinjected into GV oocytes (10m pl per oocyte) to inhibit IgG. To remove the toxic preservative within the commercial antibodies, the antibodies were buffer-exchanged into PBS/50% glycerol with a size-exclusion spin column until the original preservative-containing antibody solution was less than 10^-4^ of the final solution.

### Oocyte cDNA synthesis and amplification of IgG VDJ region

RNA extraction and cDNA synthesis were performed with NEBNext^®^ Single Cell/Low Input cDNA Synthesis and Amplification Module (Cat No.: E6421, NEB, Lpswich, MA, USA) according to the instruction from 100 oocytes. For IgG VDJ amplification, two round of PCR were done with two set of primers respectively. The primer sequences are in supplementary table 1. The DNA polymerase was Phanta high-fidelity DNA polymerase (Cat No.: P505-d1, Vazyme, Nanjing, Jiangsu, China). Then PCR products were ligated into cloning vector (Vazyme, Cat No.: C601-02) and transformed into competent DH5α, and 50 colonies (ligated cDNA from each colony corresponds to the mRNA of a single oocyte) were sent to GENEWIZ (Suzhou, Jiangsu, China) for sequencing.

### Detection of ROS generation

The ROS Assay Kit (Cat#: S0033, Beyotime, Beijing, China) was used to detect ROS generation in oocytes. In brief, oocytes were incubated with a dichlorofluorescein diacetate (DCFH-DA) probe for 20 min at 37°C in the dark, washed, and mounted on slides for confocal imaging.

### Mitochondrial staining and ATP measurements

For mitochondrial staining, oocytes were incubated in Hepes containing 100 nM Mito Tracker (Cat#: M7521, Invitrogen, Carlsbad, CA, USA) and 10 µg/ml Hochest 33342 (Sigma) for 30 min. Images were taken with an Andor Revolution spin disk confocal workstation.

For ATP measurements, 15 oocytes were first lysed with 100 µl ATP lysis solution (Cat#: S0026, Beyotime) on ice. The samples were then detected by enzyme-labeled instrument Synergy2 (BioTek, Winooski, VT, USA) to evaluate ATP level.

### Apoptosis detection

Apoptosis was detected with an Annexin V-FITC/PI Apoptosis Detection Kit (Cat#: 40302ES20, YEASEN). Oocytes were stained with 5 μl annexin V-FITC and 10 μL PI Staining Solution diluted in 100 μl binding buffer for 15 min in the dark at RT. After washing, oocytes were mounted onto glass slides, and images were obtained as above.

### Chromosome spread

Oocytes were exposed to Tyrode’s buffer (pH 2.5) for 40–50 s to remove ZP, then fixed in a drop of 1% paraformaldehyde with 0.15% Triton X-100 on a glass slide. Kinetochore and chromosome were then stained as that of immunofluorescence. The Andor Revolution spinning disk confocal workstation was used to examine chromosome and kinetochore numbers in oocytes.

### Animal/oocyte inclusion, experiment grouping, data collection, and data analysis

Any selected oocyte must be of normal quality (fully-grown oocyte from an antral follicle, normal diameter, tight connection between zona pellucida and oocyte membrane, etc). Any selected female mouse has to be physically healthy (normal body weight, eats normally, normal activity, etc). Any oocyte or mouse of bad quality or unhealthy will be excluded.

For sample size in oocyte-related experiment, about 3-5 oocytes were randomly selected for one repeat and thus about 9-15 oocytes were analyzed for three independent repeats. We found that measurements from 9-15 oocytes could form a normal distribution and are fairly reliable for a statistic analysis. For sample size in mouse-related experiment, similarly, about 3-5 mice were randomly selected for one repeat and 9-15 mice were analyzed for three independent repeats.

We tried to follow the double-blinding roles for both mouse and oocyte-related experiment grouping, data collection, and data analysis. For each independent repeat, oocytes were obtained from multiple mice to eliminate natural differences between individuals; oocytes were randomly divided into different experimental groups. About 50 oocytes per group for immunofluorescence/live fluorescence or 100 oocytes per group for western blot were used. For image taking, the order for different groups was solely random. And within the same group, oocytes were again randomly selected for image taking (We didn’t take images from all oocytes). The index setting for image taking was always identical for each group of the same experiments. The order of different groups for data measurement is also random. All intensity measurements were done with Image J on the original tif file without any adjustment of brightness/contrast. Fluorescence intensity was always a net intensity obtained by a measured image intensity subtracted by the adjacent background intensity. For band quantification in western blot, an integrated intensity was obtained by the average intensity multiplied by the band area; the average intensity was again obtained by measured image intensity subtracted by the adjacent background intensity.

### Statistical analysis

All experiments were repeated at least three times. Data are presented as mean ± SEM. Comparisons between two groups were made by Student’s t test. Differences among more than two groups were compared using one-way ANOVA. P < 0.05 was considered statistically significant. Statistical analyses were conducted with GraphPad Prism.

## Data availability statement

Chip-seq raw data have been deposited into GEO repository, the accession No. is GSE191278. **Supplementary Dataset 1** has been deposited into Zenodo, the DOI is 10.5281/zenodo.5793909.

## Ethics statement

The animal study was reviewed and approved by Animal Ethics Committee of Nanjing Medical University (NJMU) (approval no. IACUC-1809011).

## Author contributions

DZ, Y-CG, C-LZ and Z-XY designed the research. YW, F-QL and Y-HH performed most of the experiments. XW, C-RL, B-QC, L-JC and Z-BW assisted during the experiments. YW did all data analysis and prepared figures under the direction of DZ, Y-CG, C-LZ and Z-XY. DZ and Z-XY wrote the manuscript, C-LZ and Y-CG proofread and gave advice. All authors contributed to the article and approved the submitted version.

## Funding

This work is financially supported by the General Program of the National Natural Science Foundation of China (Grant No: 32070840) to DZ, Science and technology research plan of Henan Province (Grant No. 202102310061) to Y-CG and the major project of Henan Provincial medical science and technology plan (Grant No. SBGJ202001002) to C-LZ.

## Acknowledgment

We thank Professor Shuo Yang and Professor Xiao-Ming Wang from Department of Immunology, Nanjing Medical University, and Wei-Guo Zhang from Suzhou Institute of Systematic Medicine, Systematic Medical Research Center, Chinese Academy of Medical Sciences for valuable advices.

## Conflict of interest

The authors declare that the research was conducted in the absence of any commercial or financial relationships that could be construed as a potential conflict of interest.

## Publisher’s note

All claims expressed in this article are solely those of the authors and do not necessarily represent those of their affiliated organizations, or those of the publisher, the editors and the reviewers. Any product that may be evaluated in this article, or claim that may be made by its manufacturer, is not guaranteed or endorsed by the publisher.

## Abbreviations

CTCF, CCCTC-binding factor; RAG1/2, Recombination activating gene 1/2; VprBP, DDB1 and CUL4 associated factor 1; NF-kappa B, v-rel reticuloendotheliosis viral oncogene homolog A; also known as NFKB, p65; FcγR, Fc receptor; CR-1, complement receptor 2, also known as CR2; CR-3, integrin alpha M, C1q-R, CD93 antigen; LCMS, Liquid Chromatography Mass Spectrometry; SCID, server combined immune-deficiency; LPS, Lipopolysaccharide; GCs, granular cells; CD46, CD46 antigen, complement regulatory protein, FCGR1A, Fc fragment of IgG receptor Ia; CSF1, colony stimulating factor 1; NMN, Nicotinamide mononucleotide; ssRNASingle-Stranded Ribonucleic Acid, ZP,zona pellucida; IVM, *In-vitro* maturation; HIV, human immunodeficiency virus; SOD2, Superoxide dismutase 2, GPX4, Glutathione peroxidase 4.
