# Supplementary material for: Oocytes could rearrange immunoglobulin production to survive over adverse environmental stimuli
Source: Front Immunol. 2022 Nov 2;13:990077. doi: 10.3389/fimmu.2022.990077 (PMC9667025; doi:10.3389/fimmu.2022.990077)
Supplement: Supplementary file 3 [file DataSheet_2.docx]

**SUPPLEMENTARY Figure legends**

**Supplementary Figure 1. IgG transcription was not affected by H3K4me^3^ in oocytes**

H3K4me^3^ chip-seq showed that on exposure to either LPS or CSF1 stimuli, H3K4me^3^ decreased its binding to the regulating sequences of all genes; Meanwhile, its binding to the regulating sequences of the IgG gene did not show difference from control.

**SUPPLEMENTARY dataset legends**

**Supplementary Dataset 1. IgG antibody immunoprecipitation and mass-spec in oocyte lysates identified IgG fragment**

This file includes two sheets. "Peptides" showed all peptides identified through mass-spec, "Protein Groups" showed the proteins corresponding to all identified peptides; Ighv2-5 is blue-highlighted in either sheet.

**SUPPLEMENTARY movie legends**

**Supplementary Movie 1. Dynamic increment of IgG on oocyte membrane upon *E. coli* treatment**

Live imaging showed that two hours of *E. coli* stimuli, large IgG aggregates gradually increased across the whole membrane, from the inner side to outer side. Time interval, 15 min.

**Supplementary tables**

**Supplementary table 1. Primers for IgG mRNA**

**IgG-VDJ chain**

| 1st PCR IgH-VDJ^1^ | Primer sequence |
| --- | --- |
| Forward |  |
| 5’ 1mFH_I^2^ | AGGAACTGCAGGTGTCC |
| 5’ 1mFH_II^2^ | CAGCTACAGGTGTCCACTCC |
| 5’ 1mFH_III^2^ | TGGCAGCARCAGCTACAGG |
| 5’ 1mFH_IV^2^ | CTGCCTGGTGACATTCCCA |
| 5’ 1mFH_V^2^ | CCAAGCTGTGTCCTGTC |
| 5’ 1mFH_VI^2^ | TTTTAAAAGGTGTCCAGKGT |
| 5’ 1mFH_VII^2^ | CCTGTCAGTAACTRCAGGTGTCC |
| 5’ 1mFH_VIII^2^ | TTTTAAAAGGGGTCCAGTGT |
| 5’ 1mFH_IX^2^ | CGTTCCTGGTATCCTGTCT |
| 5’ 1mFH_X^2^ | ATGAAGTTGTGGYTRAACTGG |
| 5’ 1mFH_XI^2^ | TGTTGGGGCTKAAGTGGG |
| 3’ 1mRG(Gamma)^3^ | AGAAGGTGTGCACACCGCTGGAC |
|  |  |
| 2nd PCR IgH-VDJ^1^ | Primer sequence |
| 5’ 2mFG^2^ | GGGAATTCGAGGTGCAGCTGCAGGAGTCTGG |
| 3’ 2mRG(Gamma)^3^ | GCTCAGGGAARTAGCCCTTGAC |

**Constant chain**

| PCR IgH-Constant^1^ | Primer sequence |
| --- | --- |
| 5’ 2mRG(Gamma)^2^ | GTCAAGGGCTAYTTCCCTGAGC |
| 3’ IgG1^3^ | TCATTTACCAGGAGAGTGGGAG |
| 3’ IgG2a^3^ | GACCTGAGAGTTTTGTGGGTG |
| 3’ IgG2b^3^ | TCATTTACCCGGAGACCG |
| 3’ IgG3^3^ | AAGCTCTGGCACCTTCTGAAG |

^1^ Regions to be amplified.

^2^ These are forward primers.

^3^ These are reverse primers.

**Supplementary table 2. DNA oligos for ssRNA**

| **ssRNA** | **DNA templates** |
| --- | --- |
| ssRNA-6T | Oligo1: GGATCCTAATACGACTCACTATAGACTTGAGCGAGCGCTTTTTT^1^ |
|  | Oligo2: AAAAAAAAGCGCTCGCTCAAGTCTATAGTGAGTCGTATTAGGATCC^1^ |
| ssRNA-5T | Oligo1: GGATCCTAATACGACTCACTATAGTCCGGGCAGGTCTACTTTTT^1^ |
|  | Oligo2: AAAAAAAGTAGACCTGCCCGGACTATAGTGAGTCGTATTAGGATCC^1^ |

^1^ A pair of DNA oligos is needed for each single-stand RNA. Oligo 2 is complementary with oligo 1 except an "AA" overhang at 5'; In each oligo, gene-specific sequences are underlined, other sequences are for recognition and binding by T7 RNA polymerase.
