# Supplementary figures and images for: Oocytes could rearrange immunoglobulin production to survive over adverse environmental stimuli
Source: Front Immunol. 2022 Nov 2;13:990077. doi: 10.3389/fimmu.2022.990077 (PMC9667025; doi:10.3389/fimmu.2022.990077)

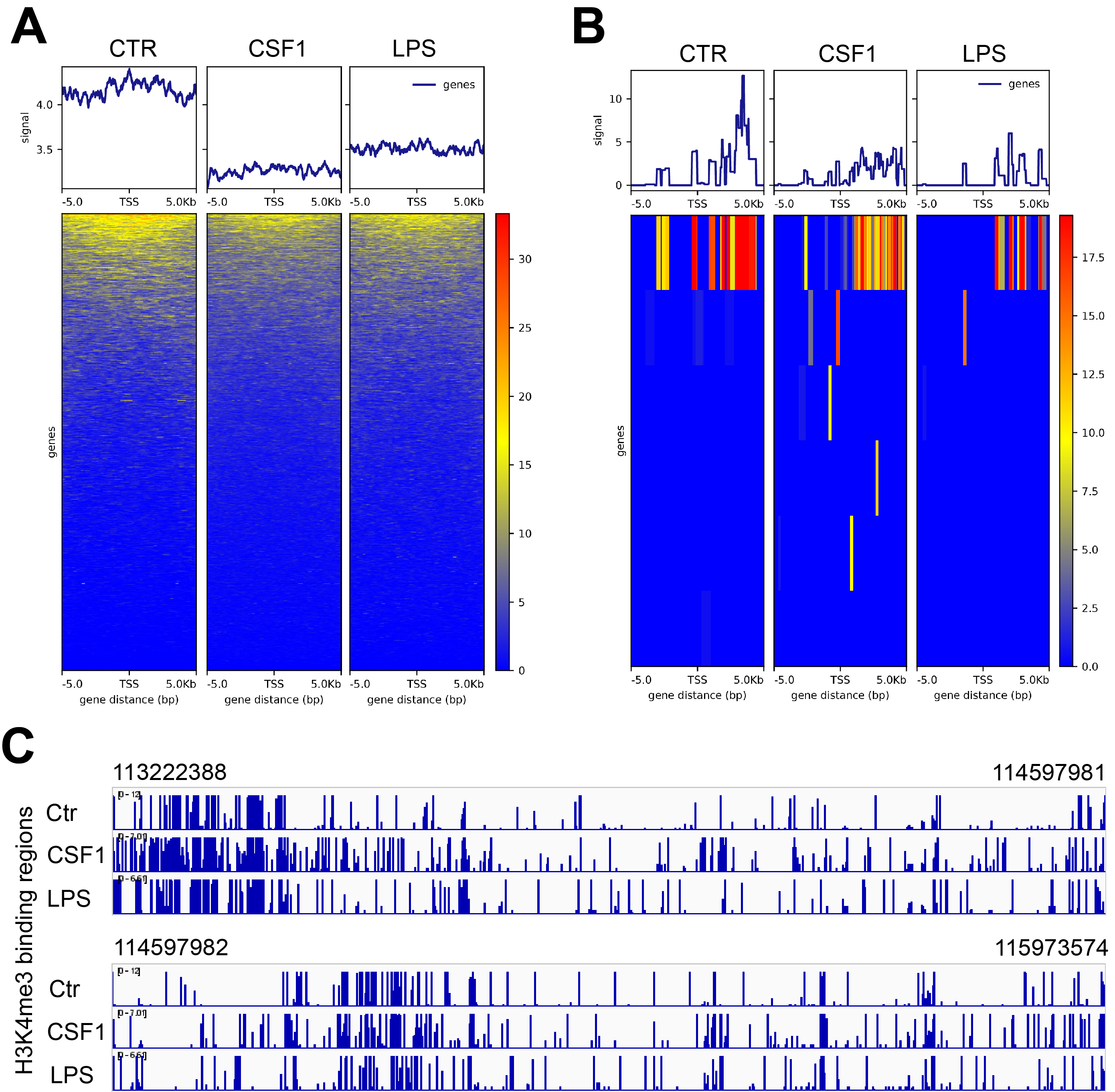

Supplement: Supplementary file 4 [file Image_1.jpeg]
